# Ovarian germ cell malignancies in England: epidemiological parallels with testicular cancer.

**DOI:** 10.1038/bjc.1991.180

**Published:** 1991-05

**Authors:** I. dos Santos Silva, A. J. Swerdlow

**Affiliations:** Department of Epidemiology and Population Sciences, London School of Hygiene and Tropical Medicine, UK.

## Abstract

The epidemiology of germ cell cancer of the ovary has been little investigated. We studied ovarian germ cell cancers incident 1971-84 in England, using data from the England and Wales national cancer register. The age distribution showed a sharp peak at ages 15-19, to which both teratomas and dysgerminomas contributed equally, and a secondary, much wider peak, at ages 65-69, mainly due to teratomas. For teratomas there were diverging secular trends by age: incidence has been increasing at ages 0-44 (P around 0.05) and decreasing at ages over 44 (P less than 0.01). Birth cohort analysis showed an increase in risk at ages 0-44 for more recent generations of women. There were no changes over time for dysgerminomas. There was no clear geographic pattern of distribution across the regions of England. The early age peak, and the increase in incidence of ovarian germ cell cancers at young ages but decrease at older ages, resembles testicular cancer epidemiology. Interestingly, discrepancies and similarities in the age distribution of these tumours between the sexes parallel lifetime profiles of gonadotropin levels in each sex.


Br. J. Cancer (1991), 63, 814-818 © Macmillan Press Ltd., 1991

### Ovarian germ cell malignancies in England: epidemiological parallels with testicular cancer

I. dos Santos Silva[1] & A.J. Swerdlow[1,2]

[1]Department of Epidemiology and Population Sciences, London School of Hygiene and Tropical Medicine, Keppel Street, London WC1 E 7HT; and [2]Office of Population Censuses and Surveys, St Catherines House, 10 Kingsway, London WC2B 6JP, UK.

**Summary** The epidemiology of germ cell cancer of the ovary has been little investigated. We studied ovarian germ cell cancers incident 1971-84 in England, using data from the England and Wales national cancer register. The age distribution showed a sharp peak at ages 15-19, to which both teratomas and dysgerminomas contributed equally, and a secondary, much wider peak, at ages 65-69, mainly due to teratomas. For teratomas there were diverging secular trends by age: incidence has been increasing at ages 0-44 ($P$ around 0.05) and decreasing at ages over 44 ($P < 0.01$). Birth cohort analysis showed an increase in risk at ages 0-44 for more recent generations of women. There were no changes over time for dysgerminomas. There was no clear geographic pattern of distribution across the regions of England. The early age peak, and the increase in incidence of ovarian germ cell cancers at young ages but decrease at older ages, resembles testicular cancer epidemiology. Interestingly, discrepancies and similarities in the age distribution of these tumours between the sexes parallel lifetime profiles of gonadotropin levels in each sex.

Germ cell cancers comprise a small proportion of all ovarian cancers (Weiss *et al.*, 1977) whereas the corresponding tumours in males account for almost all testicular cancers (Pike *et al.*, 1987). Probably as a consequence, whilst there are many population-based studies on the epidemiology of testis cancer, there is very little information available for its ovarian counterpart. To our knowledge, only one population-based study (Walker *et al.*, 1984) has assessed the time trends of these rare tumours, and then only for a short period and from a relatively small catchment population.

Early exposures in life, particularly oestrogen exposures *in utero*, have recently been investigated as potential risk factors for ovarian germ cell malignancies (Walker *et al.*, 1988). This pre-natal oestrogen hypothesis was first raised for germ cell tumours of the testis (Henderson *et al.*, 1979; Loughlin *et al.*, 1980; Schottenfeld *et al.*, 1980; Depue *et al.*, 1983) and later, extended to ovarian germ cell cancer on the assumption that if they have the same histogenesis (Fox, 1980) and similar early age peaks (Walker *et al.*, 1984), they may share at least some aetiological factors.

The England and Wales national cancer registry holds an exceptionally large cancer dataset from a catchment population of about 50 million. Tumours are coded in its files according to both site and histology (Swerdlow, 1986), although the data appear not to have been used previously for national analyses of cancer by histology. We used data from this registry to assess the epidemiological features of this female cancer and to draw comparisons which might shed light on the epidemiology of testicular cancer, the most common malignancy in young men (Davies, 1981).

### Material and methods

Cancer registration data in England and Wales are collected by regional registries. From the clinical notes, pathology records and various other sources, they extract information on the characteristics of the tumour, including its histology. Data are then sent to the national cancer registry at the Office of Population Censuses and Surveys (OPCS) who collate, analyse and publish them. Further details are given elsewhere (Swerdlow, 1986).

From the OPCS files, we extracted all cancer registrations incident 1971-84 whose primary site of malignancy was allocated to ovary (International Classification of Diseases code 183.0) (World Health Organization, 1967, 1978) and whose histology was reported to be of germ cell nature. Tumour histology was coded according to the Manual of Tumour Nomenclature and Coding (MOTNAC) (American Cancer Society, 1968) for 1971-78 data and the International Classification of Diseases for Oncology (ICD-O) (World Health Organization, 1976) for data from 1979 onwards. The present analysis was restricted to 1971 onwards, because the histological code used before then, a two-digit OPCS code, was very elementary. The analyses were all restricted to England, because of incompleteness in the Welsh data, as explained under 'Results'. We also extracted from the OPCS files: (1) tabulations of ovarian cancer registrations incident 1971-84 by histology and (2) mid-year population estimates of England and its hospital regions for 1971-84. Tabulations of testicular cancer (ICD: 186) registrations incident 1971-84 by hospital region of residence were taken from published sources (Office of Population Censuses and Surveys, 1979-1988).

Since data on histological type were incomplete in the OPCS files, the observed numbers of ovarian cancers with germ cell histology in the files were an underestimation of the true numbers of incident cases and the extent of this underestimation will vary by region and time. We therefore estimated the true numbers by multiplying, in each year of registration, region and 5-year age-group category, the observed number of registrations by the inverse of the corresponding proportion of ovarian cancers with histological confirmation. The analyses were executed both on the unadjusted data and on the estimated true incident numbers, but since they showed similar trends the results given here refer to the adjusted data unless otherwise specified.

Analyses were carried out for ovarian germ cell cancers overall and separately for its two major histological categories, dysgerminomas and teratomas. Both embryonal and extra-embryonal cell types (i.e. embryonal cell carcinoma, endodermal sinus tumour and choriocarcinoma) were included in the latter category (Fox, 1980). Directly age-standardised rates were calculated using the 1978 female mid-year population of England as the standard.

To assess secular trends of incidence, we fitted a Poisson regression model (Breslow & Day, 1987). Estimated numbers of incident cases were truncated to whole numbers before the model was fitted. Due to small numbers, time trends in specific age-groups (0-14, 15-24, ..., 65-74, 75+) were analysed by splitting the 14-year study period into three

Correspondence: I. dos Santos Silva.
Received 24 September 1990; and in revised form 2 January 1991.

calendar periods: 1971–75, 1976–80 and 1981–84; statistical significance of trends was assessed by the Mantel-Haenszel chi-square test (Breslow & Day, 1980).

To examine risks according to birth cohort, standardised cohort registration ratios (SCRRs) were calculated (Beral *et al.*, 1978), again using the female 1978 mid-year population as the standard. The SCRR summarises by direct standardisation the cumulative risk experience of each successive generation of women relative to the overall risk for all women; age-specific comparisons are cumulated up to the age which a particular generation has reached at the end of the analysis period. An SCRR of 120, for instance, would indicate that the risk for that particular generation was 20% above the average for all women of England in the analysis, whereas an SCRR of 80 would indicate that the risk was 20% below. Exact year of birth, which was known for all cases, was used in the cohort analysis. Since data on population by year of birth were not available, these were estimated from OPCS statistics on the population by calendar year and single year of age; for each age *versus* calendar year combination in these statistics, two adjacent years of birth were possible, and we assumed that the populations were born equally in these 2 years.

Geographical distribution was analysed by hospital region of residence for the study years aggregated; there were minor regional boundary changes in 1974, but their effect should have been negligible for the present analysis. For comparison, the geographical distribution of testicular cancer was also analysed for the same period. The significance of regional incidence rates compared to national rates was tested using the significance factors of a Poisson distribution (Bailar & Ederer, 1964). The 95% confidence intervals were slightly conservative since estimated numbers with decimal figures were truncated.

All calculations were carried out in the EPILOG statistical software package (EPICENTER SOFTWARE, 1985).

### Results

Table I shows percentages of ovarian tumours with histology known by region and age. There was virtually no change over time in the percentage confirmed for England overall, although some individual regions showed increases or decreases. The proportion known was greatest at younger ages: at 0–44 almost 90% were known nationally, and over 80% were known in all but three regions. At older ages the proportion decreased: at ages 65 and above, around 60% were known nationally and in most but not all regions. Wales had a very low proportion of ovarian cancers with known histology (20%), and for this reason was excluded entirely from the present study. We also excluded from the analyses three registrations (0.53%) with unknown hospital region of residence, leaving a total of 558 incident cases.

These represented 1.4% of all ovarian cancers of known histology registered from the same population. Teratomas comprised 63.8% (356) of these tumours and dysgerminomas 36.2% (202).

The age distribution of ovarian germ cell cancer for the study period (Figure 1) showed an early sharp peak at ages 15–19, to which both histologies contributed equally, and a later, but broader peak at ages 65–69, mainly due to teratomas. Analysis by single year (Table II) showed that teratomas peaked slightly earlier (at age 17) than dysgerminomas (at age 19).

Secular trends in incidence of ovarian germ cell cancer by cell type and age are shown in Figures 2 and 3. There was no significant linear trend for germ cell cancer overall ($b = 0.024$, $P = 0.80$) in England (Figure 2b) or any of the regions examined separately (not shown in Figure), although there was some decrease in incidence rates since 1979. Again, no significant trend emerged when analysis was subdivided by cell type (Figure 2b) (teratomas: $b = -0.006$, $P = 0.61$; dysgerminomas: $b = 0.002$, $P = 0.90$). However, analyses by age and histology showed that there were diverging time trends in teratoma incidence for young and for older women (Figure 3): at ages under 45, there was a significant increase in incidence ($b = 0.035$, $P = 0.04$), whereas at ages 45 and over there was a significant decrease ($b = -0.053$, $P = 0.003$). Dysgerminoma rates have remained constant over time in both age-groups (at 0–44 years, $b = -0.006$, $P = 0.76$; at 45 + years, $b = -0.082$, $P = 0.31$). Ovarian germ cell cancer overall showed similar diverging trends by age to teratomas, but only significant in older women ($b = -0.04$; $P = 0.01$). There was no correlation between teratoma and dysgerminoma rates by year ($r = 0.10$, $P = 0.74$).

Further breakdown by age, showed that the increase in teratoma incidence occurred across all groups under age 45 and the decrease across all groups older than this; the trends were significant only at ages 55–64 ($\chi^2_1 = 3.88$; $P = 0.048$)

**Figure 1** Age distribution of ovarian germ cell cancer in England. Mean annual registration rates for 1971–84.

**Table I** Proportions of registered ovarian cancers with histology known, by region and age, England 1971–84

| *Region* | *0–44* | *45–64* | *65 +* | *All ages* |
|---|---|---|---|---|
| | *Age (years)* | | | |
| Northern | 82.8 | 81.5 | 62.8 | 73.83 |
| York | 86.5 | 79.9 | 62.7 | 73.08 |
| Trent | 75.2 | 67.1 | 48.7 | 60.24 |
| E Anglia | 84.2 | 79.2 | 61.7 | 71.61 |
| NW Thames | 81.6 | 72.0 | 56.8 | 66.52 |
| NE Thames | 72.6 | 60.8 | 44.9 | 54.61 |
| SE Thames | 87.4 | 82.6 | 64.1 | 74.07 |
| SW Thames | 86.2 | 83.4 | 67.5 | 76.02 |
| Wessex | 52.8 | 46.9 | 37.7 | 43.30 |
| Oxford | 86.5 | 86.0 | 74.8 | 81.32 |
| S Western | 81.9 | 75.7 | 61.6 | 69.52 |
| W Midlands | 85.8 | 82.0 | 66.1 | 76.12 |
| N Western | 87.9 | 76.8 | 59.6 | 69.74 |
| Mersey | 88.5 | 77.7 | 60.5 | 71.40 |
| England | 88.5 | 77.7 | 60.5 | 68.71 |

**Table II** Age distribution of ovarian germ cell cancers of known histology, England 1971–84

| *Age* | *Teratomas Rate* (No)* | *Dysgerminomas Rate* (No)* | *Age* | *Teratomas Rate* (No)* | *Dysgerminomas Rate* (No)* |
|---|---|---|---|---|---|
| 0- | 0.00 (0) | 0.00 (0) | 13- | 1.29 (8) | 1.29 (8) |
| 1- | 0.53 (2) | 0.00 (0) | 14- | 2.15 (10) | 0.43 (2) |
| 2- | 0.26 (1) | 0.00 (0) | 15- | 1.51 (7) | 1.51 (7) |
| 3- | 0.19 (1) | 0.00 (0) | 16- | 1.52 (7) | 1.51 (7) |
| 4- | 0.25 (1) | 0.00 (0) | 17- | 2.62 (12) | 2.19 (10) |
| 5- | 0.00 (0) | 0.00 (0) | 18- | 2.14 (13) | 2.14 (13) |
| 6- | 0.00 (0) | 0.00 (0) | 19- | 1.78 (8) | 2.45 (11) |
| 7- | 0.47 (2) | 0.24 (1) | 20- | 1.58 (7) | 2.25 (10) |
| 8- | 0.00 (0) | 0.16 (1) | 21- | 1.60 (7) | 1.60 (7) |
| 9- | 1.12 (5) | 0.45 (2) | 22- | 1.38 (6) | 1.15 (5) |
| 10- | 0.66 (3) | 1.32 (6) | 23- | 1.15 (7) | 1.98 (12) |
| 11- | 1.30 (6) | 1.08 (5) | 24- | 0.70 (3) | 1.63 (7) |
| 12 | 0.86 (4) | 1.50 (7) | 25- | 1.15 (5) | 1.61 (7) |

*Rate per million.

**Figure 2** Secular trends in ovarian germ cell cancer incidence by cell type, England, 1971–84 (annual age-standardised rates): before **a** and after **b** adjustment for cases of unknown histology (see text).

**Figure 3** Secular trends and linear regression lines of ovarian malignant teratomas by age, England, 1971–84 (annual age-standardised rates).

and 75 and over ($\chi^2_1$ = 5.22; $P$ = 0.02). Dysgerminomas showed an irregular pattern across the different age-groups; there was a significant increase in incidence at ages 55–64 ($\chi^2_1$ = 4.84; $P$ = 0.03) but based on small numbers (one case in 1971–75). The above analyses were adjusted for cases of histology not known, but repetition using only actual numbers of cases with histology known gave similar results (Figure 2a): teratomas showed diverging trends, although only significant in older women (ages 0–44: $b$ = 0.031, $P$ = 0.07; ages 45+: $b$ = −0.047, $P$ = 0.01).

Analysis by birth cohort (Figure 4) showed a slight increase in risk of ovarian germ cell cancer before age 45 years for the most recent generations of women ($b$ = 2.93, $P$ = 0.01), particularly for teratomas ($b$ = 5.57, $P$ = 0.01); there was no change for dysgerminomas. The risk in women aged 45 and above remained constant. Again, the unadjusted analysis showed similar cohort trends (for ages under 45, all germ cell cancers: $b$ = 2.99, $P$ = 0.03; teratomas: $b$ = 5.48, $P$ = 0.03).

**Figure 4** Incidence trends by birth cohort of ovarian germ cancer among women aged 0–44 years born 1935–1969, England.

Analysis by hospital region of residence age 0–44 years did not show any clear geographic pattern: East Anglia and Trent had the highest rates (3.47 and 2.89 per million per annum, respectively), but only significantly raised compared to England for the former region ($P$<0.05). There was also no correlation between teratoma and dysgerminoma risk over the regions of England ($r$ = 0.12, $P$ = 0.69), nor between ovarian germ cell cancer overall ($r$ = 0.27, $P$ = 0.35) or any of its cell types and testicular cancer risk across regions. The same picture emerged from repitition of these analyses with the unadjusted data.

### Discussion

Our data suggested that ovarian germ cell malignancies comprise two different epidemiological entities: the first occurring at young ages and the second at older ages, with a division at around 45 years. The young peak, a typical feature of these tumours, was formed equally by dysgerminomas and teratomas; in our data the peak was slightly younger for teratomas than dysgerminomas, as in other populations (Stalsberg *et al.*, 1983; Walker *et al.*, 1984). The peak at older ages was largely formed by teratomas, being much more prominent in the present data than in Los Angeles (Walker *et al.*, 1984), but similar to that reported from several countries by Stalsberg *et al.* (1983) and the US Third National Cancer Survey 1969–71 (Weiss *et al.*, 1977). The early age peak of ovarian germ cell malignancies resembles that of testicular germ cell cancer, except that it occurs about 10 years earlier; as seen in females, teratomas in males peak earlier than seminomas (the equivalent of dysgerminomas in females) (Walker *et al.*, 1984; Pike *et al.*, 1987). Testicular cancer has a small peak in children under 5 years, although only in part of germ cell origin (Pike *et al.*, 1987); our data were insufficient to examine the distribution in childhood (only five cases occurred under age 5 years) but data from the Oxford Childhood Cancer Survey, a larger dataset from all Britain, suggest that there may well be a peak around age 2 (G.J. Draper, personal communication). The peak at older ages for ovarian cancer has no counterpart in males: there is a rise in incidence of cancer of the testis at older ages, but it is not of germ cell histology (Pike *et al.*, 1987).

Time trends in ovarian germ cell cancer were different for the two main age-groups, resulting from differences for teratomas: a rise in incidence was observed in young women, although only statistically significant for the adjusted data, and a decline in older women. Analysis by birth cohort also implied an increasing risk for more recent generations of women aged 0–44. The observed SCRRs were more accurate than usual; use of exact year of birth avoided the overlap between adjacent generations which occurs with the conventional method of Case (1956). Since the overall, all-years, data had to be used to calculate the expected values, the ratio of observed to expected will tend to have been conservatively biased, particularly in the earliest and latest cohorts, and therefore real changes might have been underestimated. Interpretation should also take into account that the number of cases by year of birth was small, particularly for dysgerminomas, and that the experience for the most recent generations was based only on the (young) age-groups that they have yet reached. Walker *et al.* (1984) also reported an increasing secular trend in ovarian germ cell cancer in young women (although mainly in dysgerminomas) and no significant changes at older ages, but their analyses were based on very few cases. This contrasts with a relatively constant mortality from these tumours observed during the fifties and sixties in the United States (Li *et al.*, 1973). An increase in incidence at young ages and a decrease at older ages with cohort effects underlying them has been shown in males for testicular cancer (Davies, 1981; Osterlind, 1986).

Potential artifacts need to be considered. Completeness of cancer registration in England has probably improved over time (Swerdlow, 1986), but this seems unlikely to have distorted the results since the regional analyses showed results for registries whose completeness appears to have been very high throughout the period similar to those observed for England overall. Potential late registrations, yet to be entered onto the OPCS data files, are likely to have been of negligible effect since the analyses were conducted only to 1984, 7 years ago. The decrease in rates since 1979, in particular, is unlikely to be due to this, since we have calculated rates for ovarian cancer overall and they do not show any downward trend in recent years.

Incompleteness of histological confirmation seems unlikely to explain the results since the proportion of ovarian cancers with unknown histology was small at younger ages, when most of the tumours occurred, and the results were similar when restricted to tumours of known histology. Lack of uniform criteria among pathologists, and potential changes in diagnostic criteria over time, might have affected the results; in particular, the adjustment made for cases of unknown histology depends on the assumption that the proportions with histology known for germ cell tumours were the same as those for all ovarian cancers, which might not have been the case. Since most of the tumours occurred at young ages they were, however, more likely to be properly diagnosed. Besides, the age distribution curves from the present data were similar to those from other registry-based studies in which histology was specially reviewed (Stalsberg *et al.*, 1983).

The age distribution of ovarian germ cell malignancies did not parallel gonadal activity: although the rise in incidence from pubertal to young adult ages occurred at a time of increase in ovarian activity, the decrease observed thereafter occurred when the ovaries are functionally highly active, and the peak at older ages when their activity is greatly reduced.

There are parallels however between germ cell cancer incidence and gonadotrophic hormone levels (follicular stimulating hormone (FSH) and luteinising hormone (LH)). These hormones are known to promote the multiplication of ovarian germ cells (Henderson *et al.*, 1982). In both sexes, there is a rise in the baseline plasma levels of gonadotrophins at prepubertal ages, just before the occurrence of the young age peak in germ cell cancer, followed by a decline after puberty (Coble *et al.*, 1969; Baker *et al.*, 1976). At a later stage, due to loss of negative feedback from ovarian hormones, the onset of the menopause is accompanied by a 15-fold increase in the production of FSH and a 5-fold increase in the production of LH (Coble *et al.*, 1969; Cooke *et al.*, 1976), a situation that has no parallel in males, in whom gonadotropin levels rise only slightly after the sixth decade of life (Baker *et al.*, 1976). These sex differences in gonadotropin stimulation might be responsible for the sex discrepancy in the age distribution of these tumours, namely, the presence of the second later peak in females and not in males. Also, the fact that the young peak occurred earlier in females than males might in part be due to an earlier mean age of puberty in females (Marshall & Tanner, 1970) (although a shorter induction period after puberty would also need to be postulated to explain the full extent of the difference). High levels of plasma gonadotropins, greater even than at prepubertal ages, are present in both sexes in the first 2 years of life, decreasing thereafter (Faiman & Winter, 1971; Forest *et al.*, 1974). This is consistent with the male childhood peak. In females, a small peak of ovarian germ cell cancers was present in children in our data, but numbers were insufficient to be confident that it is real. Analyses of other germ cell cancer datasets may clarify this issue.

Gonadotropins might relate to germ cell malignancies in two ways. First, prolonged stimulation by these hormones causes ovarian tumourigenesis in animals (Murphy & Beamer, 1973) and might have a similar role in humans. Second, gonadotropins might promote the multiplication of cells that have already suffered malignant transformation (Henderson, 1982). The initiation of malignancy might occur from prenatal hormone exposure (Walker *et al.*, 1988): it is notable that the adult peak closely resembles the incidence curve of vaginal adenocarcinoma resulting from maternal exposure to diethylstilboestrol (DES) (Herbst *et al.*, 1971).

Inter-country variations in testicular cancer rates are mainly a result of differences in the adult peak (Swerdlow, 1985). Very few data are available to determine whether the same is true for germ cell cancer in females. The US Third National Cancer Survey 1969–71 (Weiss *et al.*, 1977) shows an age curve similar in rates and shape to the present one from England. In Los Angeles (Walker *et al.*, 1984), the overall incidence is higher than in England, with the excess accounted for by a more prominent young age peak, and rates at older ages indeed lower than in England. Data on ovarian cancer incidence in *Cancer Incidence in Five Continents* are only available by histology in Volume II (Doll *et al.*, 1970), and for most registries analysis is not possible because of very small numbers. In Sweden, 1962–65, the overall incidence is higher than in our data (age-standardised incidence rate of 3.72 per million per annum); there is a bimodal distribution, with rates higher than in England in all age-groups particularly older ages. Aggregating data for the four English registries included in that publication (Doll *et al.*, 1970), showed age-specific rates similar at both age peaks to the ones reported in the present data.

Perhaps variations in the use of postmenopausal oestrogen replacement therapy and in prevalence of oophorectomy might have contributed to the differences between populations in ovarian germ cell cancer risk at older ages, and the secular decrease in incidence observed in our data for women aged 45 years and over. Noncontraceptive oestrogens reduce the elevated gonadotropin plasma levels characteristic of postmenopausal women (Cooke *et al.*, 1976). It would appear worthwhile to investigate further the relation of gonadal germ cell cancers in each sex with gonadotropin levels and with administration of exogenous hormones, notably hormone replacement therapy, which affect gonadotropin levels, and with age at menarche and menopause.

We thank the Cancer Research Campaign for support of Dr Silva's work and Mrs T. Buckett for help in the extraction of data.

### References

AMERICAN CANCER SOCIETY (1968). *Manual of Tumour Nomenclature and Coding*. American Cancer Society: New York.

BAILAR, J.C. III & EDERER, F. (1964). Significance factors for the ratio of a Poisson variable to its expectation. *Biometrics*, **20**, 639.

BAKER, H.W., BURGER, H.G., DEKRETSER, D.M. & others (1976). Changes in the pituitary-testicular system with age. *Clin. Endocrinol.*, **5**, 349.

BERAL, V., FRASER, P. & CHILVERS, C. (1978). Does pregnancy protect against ovarian cancer? *Lancet*, **i**, 1083.

BRESLOW, N.E. & DAY, N.E. (1980, 1987). *Statistical Methods in Cancer Research. Vol. I – The Analysis of Case-Control Studies; Vol. II – The Design and Analysis of Cohort Studies.* Vol I: p. 130 and Vol II: p. 136. International Agency for Research on Cancer: Lyon.

CASE, R.A.M. (1956). Cohort analysis of mortality rates as an historical or narrative technique. *Br. J. Prev. Soc. Med.*, **10**, 1959.

COBLE, Y.D., KOHLER, P.O., CARGILLE, C.M. & ROSS, G.T. (1969). Production rates and metabolic clearance rates of human follicle stimulating hormone in pre-menopausal and post-menopausal women. *J. Clin. Invest.*, **48**, 359.

COOKE, I.D., ANDERTON, K.J., LENTON, E. & BURTON, M. (1976). Hormone patterns at the climateric. *Postgrad. Med. J.*, **52** (Suppl. 6), 12.

DAVIES, J.M. (1981). Testicular cancer in England and Wales: some epidemiological aspects. *Lancet*, **i**, 928.

DEPUE, R., PIKE, M. & HENDERSON, B. (1983). Estrogen exposure during gestation and risk of testicular cancer. *J. Natl Cancer Inst.*, **71**, 1151.

DOLL, R., MUIR, C., WATERHOUSE, J. (1970). (eds) *Cancer Incidence in Five Continents*. Vol. II. International Union Against Cancer: Geneva.

EPICENTER SOFTWARE (1985). *EPILOG*. Epicenter Software: Pasadena, California.

FAIMAN, C. & WINTER, J.S.D. (1971). Sex differences in gonadotropin concentrations in infancy. *Nature*, **232**, 130.

FOREST, M.G., SIZONENKO, P.C., CATHIARD, A.M. & BERTRAND, J. (1974). Hypophyso-gonadal function in humans during the first year of life. I. Evidence for testicular activity in early infancy. *J. Clinical Invest.*, **53**, 819.

FOX, H. (1980). Human ovarian tumours: classification, pathogenesis, and criteria for experimental models. In *Biology of Ovarian Neoplasia*, Murphy, E.D. & Beamer, W.G. (eds). UICC Technical Report, 11. International Union Against Cancer: Geneva.

HENDERSON, B., BENTON, B., JING, J., YU, M. & PIKE, M. (1979). Risk factors for cancer of the testis in young men. *Int. J. Cancer*, **23**, 598.

HENDERSON, B., ROSS, R., PIKE, M. & CASAGRANDE, J. (1982). Endogenous hormones as a major factor in human cancer. *Cancer Res.*, **42**, 3232.

HERBST, A.L., ULFELDER, H. & POSKANZER, D.C. (1971). Adenocarcinoma of the vagina. Association of maternal stilbestrol therapy with tumour appearance in young women. *N. Engl. J. Med.*, **284**, 878.

LI, F.P., FRAUMENI, J.F. & DALAGER, N. (1973). Ovarian cancer in the young: epidemiological observations. *Cancer*, **32**, 969.

LOUGHLIN, J.E., ROBBOY, S.J. & MORRISON, A.S. (1980). Risk factors for cancer of the testis. *N. Engl. J. Med.*, **303**, 112.

MARSHALL, W.A. & TANNER, J.M. (1970). Variations in the pattern of pubertal changes in boys. *Arch. Dis. Childhood*, **45**, 13.

MURPHY, E.D. & BEAMER, W.G. (1973). Plasma gonadotropin levels during early states of ovarian tumorigenesis in mice of the $W^x/W^v$ genotype. *Cancer Res.*, **33**, 721.

OFFICE OF POPULATION CENSUSES AND SURVEYS (1979–88). *Cancer Statistics Registration*. Series MB1 Nos 1, 2, 4, 5, 7, 8, 10–16, London: HMSO.

ØSTERLIND, A. (1986). Diverging trends in incidence and mortality of testicular cancer in Denmark, 1943–82. *Br. J. Cancer*, **53**, 501.

PIKE, M.C., CHILVERS, C.E.D. & BOBROW, L.G. (1987). Classification of testicular cancer in incidence and mortality statistics. *Br. J. Cancer*, **56**, 83.

SCHOTTENFELD, D., WARSHAUER, M., SHERLOCK, S., ZAUBER, A., LEDER, M. & PAYNE, R. (1980). The epidemiology of testicular cancer in young adults. *Am. J. Epidemiol.*, **112**, 232.

STALSBERG, H., BJARNASON, O., CARVALHO, A.R.L. & others (1983). International comparisons of histologic types of ovarian cancer registry material. In *An International Survey of Distributions of Histologic Types of Tumours of the Testis and Ovary*, Stalsberg, H. (ed.). UICC Technical Report No. 75, p. 247. International Union Against Cancer: Geneva.

SWERDLOW, A.J. (1985). Recent findings in the epidemiology of testicular cancer. In *Germ Cell Tumours II*, Jones, W.G., Ward, A.M. & Anderson, C.K. (eds). p. 101. Advances in the Biosciences, Vol. 55. Pergamon Press: Oxford.

SWERDLOW, A.J. (1986). Cancer registration in England and Wales: some aspects relevant to interpretation of the data. *J. R. Statist. Soc.*, **149**, 146.

WALKER, A.H., ROSS, R.K., PIKE, M.C. & HENDERSON, B.E. (1984). A possible rising incidence of malignant germ cell tumours in young women. *Br. J. Cancer*, **49**, 669.

WALKER, A.H., ROSS, R.K., HAILE, R.W.C. & HENDERSON, B.E. (1988). Hormonal factors and risk of ovarian germ cell cancer in young women. *Br. J. Cancer*, **57**, 418.

WEISS, N.S., HOMOCHUK, T. & YOUNG, J.C. (1977). Incidence of histologic types of ovarian cancer: the US Third National Cancer Survey, 1969–1971. *Gynecol. Oncol.*, **5**, 161.

WORLD HEALTH ORGANIZATION (1967, 1978). *Manual of the International Statistical Classification of Diseases, Injuries and Causes of Death.* Eight and Ninth Revision. World Health Organization: Geneva.

WORLD HEALTH ORGANIZATION (1976). *International Classification of Disease for Oncology*. World Health Organization: Geneva.


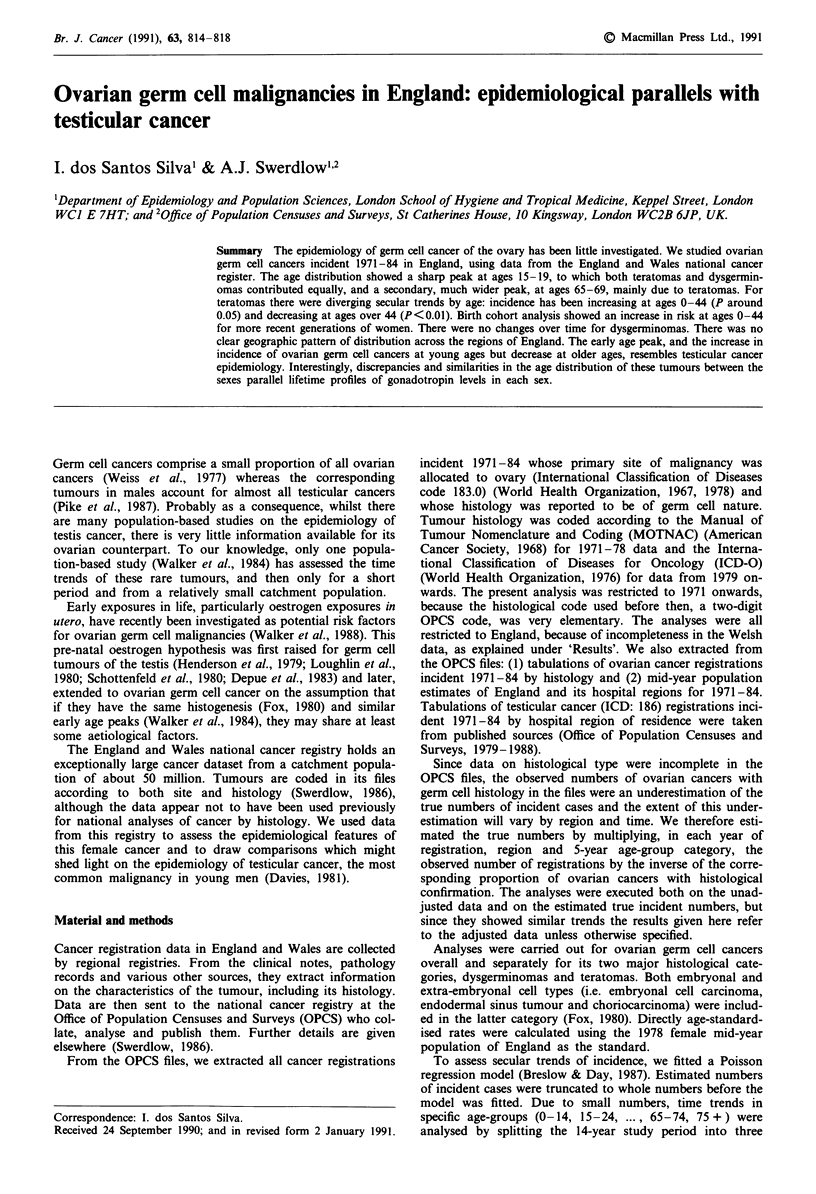

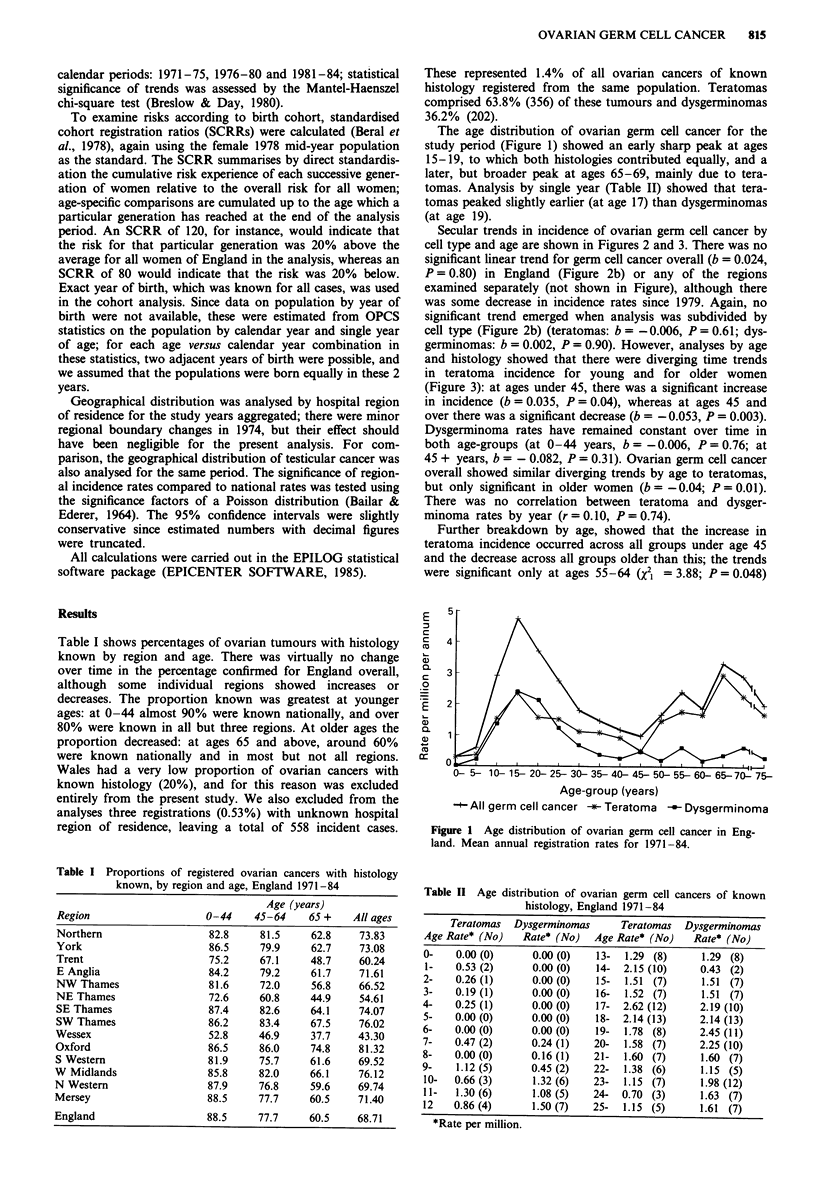

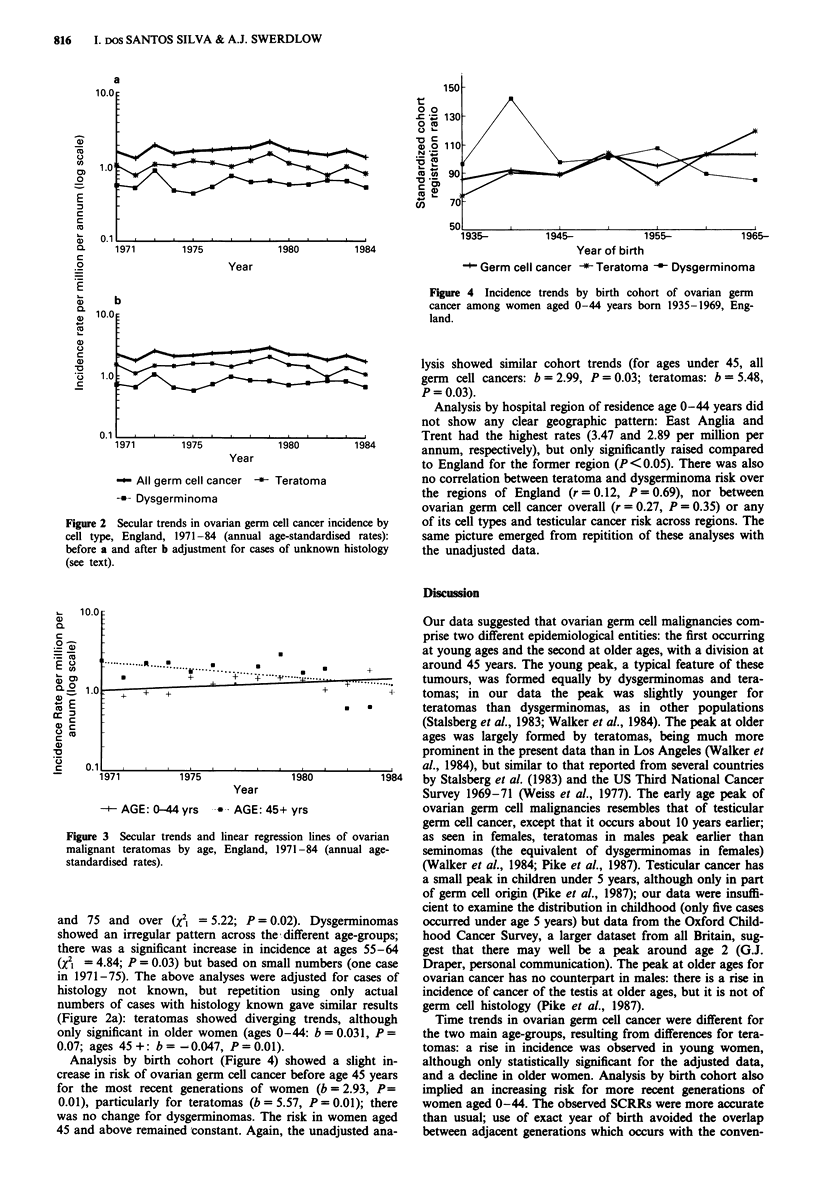

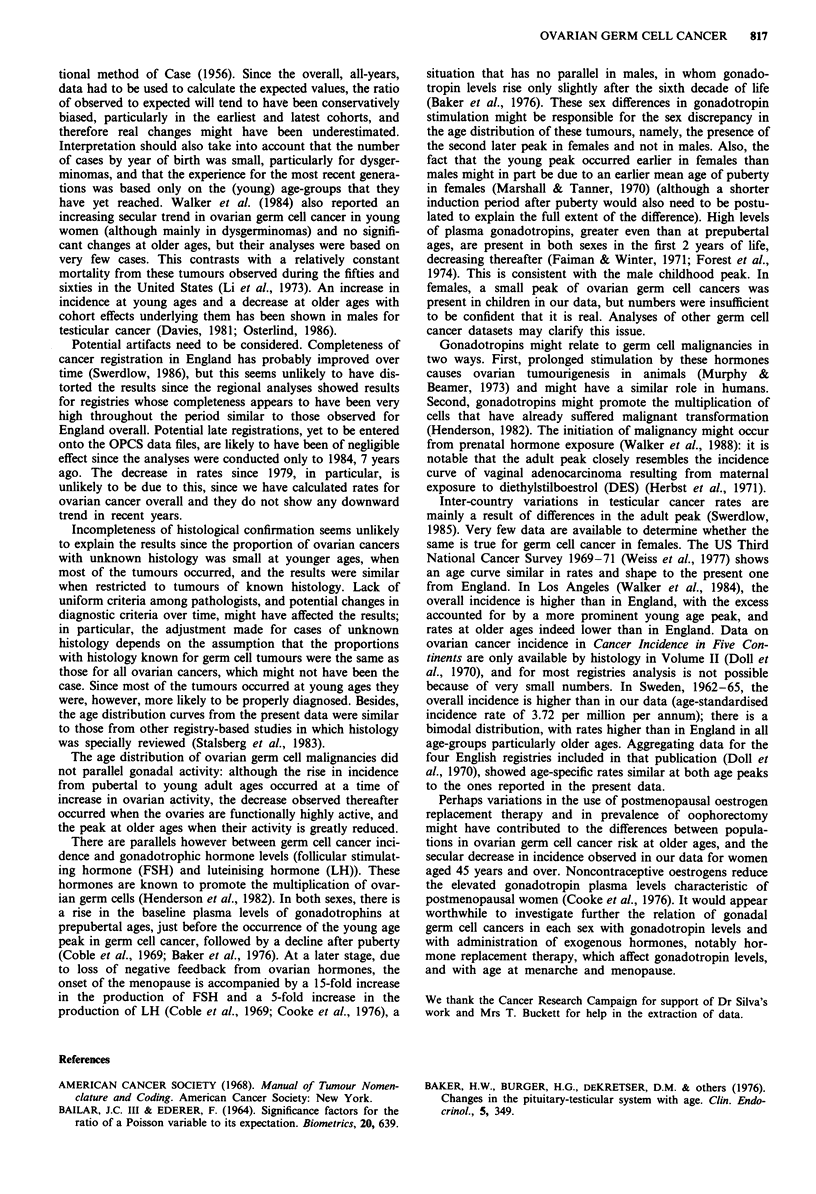

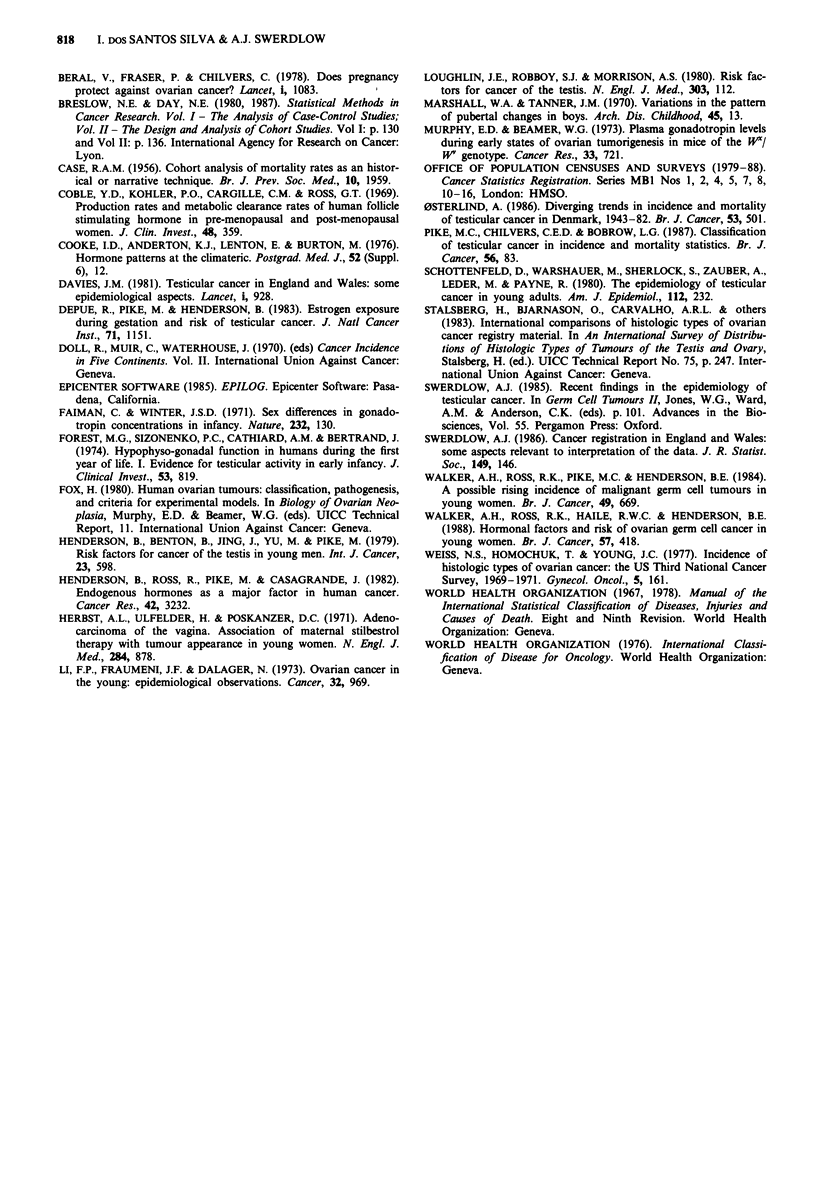


## References

[OCR_00676] Baker H. W., Burger H. G., de Kretser D. M., Hudson B., O'Connor S., Wang C., Mirovics A., Court J., Dunlop M., Rennie G. C. (1976). Changes in the pituitary-testicular system with age.. Clin Endocrinol (Oxf).

[OCR_00681] Beral V., Fraser P., Chilvers C. (1978). Does pregnancy protect against ovarian cancer?. Lancet.

[OCR_00695] Coble Y. D., Kohler P. O., Cargille C. M., Ross G. T. (1969). Production rates and metabolic clearance rates of human follicle-stimulating hormone in premenopausal and postmenopausal women.. J Clin Invest.

[OCR_00701] Cooke I. D., Anderton K. J., Lenton E., Burton M. (1976). Hormone patterns at the climacteric.. Postgrad Med J.

[OCR_00706] Davies J. M. (1981). Testicular cancer in England and Wales: some epidemiological aspects.. Lancet.

[OCR_00710] Depue R. H., Pike M. C., Henderson B. E. (1983). Estrogen exposure during gestation and risk of testicular cancer.. J Natl Cancer Inst.

[OCR_00724] Faiman C., Winter J. S. (1971). Sex differences in gonadotrophin concentrations in infancy.. Nature.

[OCR_00728] Forest M. G., Sizonenko P. C., Cathiard A. M., Bertrand J. (1974). Hypophyso-gonadal function in humans during the first year of life. 1. Evidence for testicular activity in early infancy.. J Clin Invest.

[OCR_00740] Henderson B. E., Benton B., Jing J., Yu M. C., Pike M. C. (1979). Risk factors for cancer of the testis in young men.. Int J Cancer.

[OCR_00745] Henderson B. E., Ross R. K., Pike M. C., Casagrande J. T. (1982). Endogenous hormones as a major factor in human cancer.. Cancer Res.

[OCR_00750] Herbst A. L., Ulfelder H., Poskanzer D. C. (1971). Adenocarcinoma of the vagina. Association of maternal stilbestrol therapy with tumor appearance in young women.. N Engl J Med.

[OCR_00756] Li F. P., Fraumeni J. F., Dalager N. (1973). Ovarian cancers in the young. Epidemiologic observations.. Cancer.

[OCR_00760] Loughlin J. E., Robboy S. J., Morrison A. S. (1980). Risk factors for cancer of the testis.. N Engl J Med.

[OCR_00764] Marshall W. A., Tanner J. M. (1970). Variations in the pattern of pubertal changes in boys.. Arch Dis Child.

[OCR_00768] Murphy E. D., Beamer W. G. (1973). Plasma gonadotropin levels during early stages of ovarian tumorigenesis in mice of the W x -W u genotype.. Cancer Res.

[OCR_00778] Osterlind A. (1986). Diverging trends in incidence and mortality of testicular cancer in Denmark, 1943-1982.. Br J Cancer.

[OCR_00782] Pike M. C., Chilvers C. E., Bobrow L. G. (1987). Classification of testicular cancer in incidence and mortality statistics.. Br J Cancer.

[OCR_00786] Schottenfeld D., Warshauer M. E., Sherlock S., Zauber A. G., Leder M., Payne R. (1980). The epidemiology of testicular cancer in young adults.. Am J Epidemiol.

[OCR_00815] Walker A. H., Ross R. K., Haile R. W., Henderson B. E. (1988). Hormonal factors and risk of ovarian germ cell cancer in young women.. Br J Cancer.

[OCR_00810] Walker A. H., Ross R. K., Pike M. C., Henderson B. E. (1984). A possible rising incidence of malignant germ cell tumours in young women.. Br J Cancer.

[OCR_00820] Weiss N. S., Homonchuk T., Young J. L. (1977). Incidence of the histologic types of ovarian cancer: the U.S. Third National Cancer Survey, 1969-1971.. Gynecol Oncol.

